# Supplementary material for: The stability of multitrophic communities under habitat loss
Source: Nat Commun. 2019 May 24;10:2322. doi: 10.1038/s41467-019-10370-2 (PMC6534601; doi:10.1038/s41467-019-10370-2)
Supplement: Supplementary file 1 — Supplementary Information [file 41467_2019_10370_MOESM1_ESM.pdf]

Supplementary Information

The stability of multitrophic communities under habitat loss

Chris McWilliams, Miguel Lurgi, Jose M Montoya, Alix Sauve & Daniel Montoya

Supplementary Note 1. Full specification of the individual-based model.

1. Individual-based model

Community dynamics are simulated using a spatially explicit, individual-based model (IBM) that was developed by Lurgi et al. (2016)[1]. The landscape consists of a homogeneous two-dimensional lattice (200×200 cells) on which individuals move around and interact subject to bio-energetic constraints. To avoid edge effects during the simulations, we used periodic boundary conditions, i.e. the lattice has periodic boundary conditions such that the topology of the landscape is toroidal. Each lattice cell has a space for an inhabitant and a visitor, such that a cell may contain at most two species. Basal species may only occupy the inhabitant space, whilst all other species may occupy either or both spaces. For simplicity, available, non-destroyed cells do not differ in habitat quality. Distance on the lattice is defined as follows. The immediate neighbors of any given cell are the eight adjacent cells, including diagonals (i.e. a Moore neighborhood). These eight neighbors are distance 1 from the central cell, whilst the sixteen cells surrounding them are distance 2, and so on (see Supplementary Figure 1). This distance metric is used in the rules for movement and reproduction (section 1.1, below), and in the habitat loss algorithms (section 2, below).

The model has a large parameter space - there are seventeen free parameters, which are defined in Supplementary Table 1. A discussion of the values chosen for these parameters can be found in section 1.2. Initial conditions are defined randomly by the following procedure. For each cell in the landscape an individual belonging to a randomly selected basal species is placed in the

Supplementary Figure 1. Individual movement trajectories across the simulated landscape.

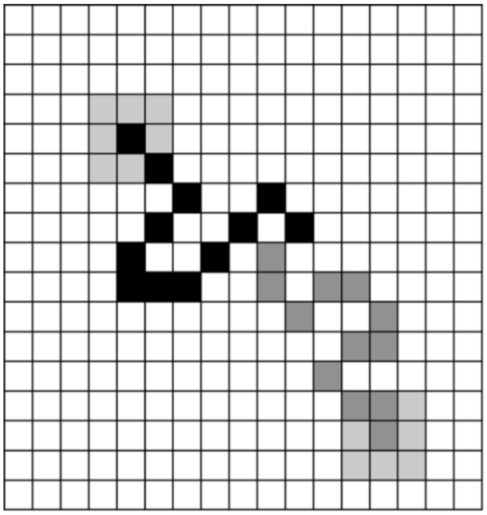

The trajectories of two individuals over 12 time steps are shown in black and dark grey. The distance-1 neighborhoods of the two individuals on the first time step are shown in light grey. Figure reproduced from[1].

inhabitant space, so that all cells contain a plant individual. Then individuals from randomly selected non-basal species are placed in the visitor space of randomly selected cells, until the desired fraction of

the landscape (given by parameter *OCCUPIED_CELLS*) is filled with animal individuals. The simulation is then run for a given number of time steps following the local rules described in section 1.1 below.

1.1 Local rules

The following local rules define the behavior of individuals, which together generate the global dynamics of the IBM. In what follows capitalized - italicized words refer to model parameters, which are defined in Supplementary Table 1. Each individual stores energy (or resource), which it expends to perform actions. Initially all individuals are given a random amount of energy between *MIN_RESOURCE* and *MAX_RESOURCE*. If the energy of an individual drops below *MIN_RESOURCE* it dies and is removed from the landscape. We assume consumers always take resource. This is a realistic assumption as all individuals are subject to energy expenditure in each time step. Thus, satiety is not considered. On each time step an initial cell is randomly selected and all cells are updated sequentially, starting at the initial cell. Cell update consists of the following ordered processes which occur first for the visitor individual and then for the inhabitant (state variables are updated asynchronously):

1. Immigration
2. Death
3. Movement
4. Reproduction
5. Feeding
6. Metabolic loss

1) Immigration

An immigrant individual is created with probability given by *IMMIGRATION*. The species of the immigrant is selected uniformly at random from the original species pool. There must be space in the cell for the immigrant to be placed, or the immigrant must be able to feed upon the species present in the cell (in which case it does so and replaces it). Otherwise the immigrant is discarded. If placed, the immigrant is given a random starting energy. All individuals bear the same immigration potential; yet, the outcome of immigration does vary across cells. This translates both into intra- and inter-specific variability in local immigration rates.

2) Death

If the energy of an individual in the cell has fallen below *MIN_RESOURCE*, it is removed from the landscape.

3) Reproduction

An individual may only reproduce if its stored energy is greater than *MATING_RESOURCE*. This is true for all species. Animals reproduce sexually, plants reproduce asexually.

- **Sexual reproduction**: If an individual's energy exceeds *MATING_RESOURCE* it searches its distance-3 neighborhood. If it finds an individual of the same species, with sufficient energy to mate, and it finds a destination cell with space for an animal (inhabitant or visitor space), then mating occurs. Both parents give a fraction of their stored energy (*MATING_ENERGY*) to the offspring, which is placed in the destination cell. If an individual has reproduced it carries out no further actions on that time step.

- **Asexual reproduction**: This occurs in basal species (i.e., plants) via two potential mechanisms of asexual reproduction: (1) 'Wind dispersal' and (2) Mutualistic dispersal:
  1. If the individual is a non-mutualistic plant it reproduces with a probability equal to *REPRODUCTION_RATE*. If reproduction occurs the offspring is placed in a randomly selected available cell in the distance-3 neighborhood. For plants, available means empty or only occupied by an animal individual. If no cells are available the plant cannot reproduce. Again, a fraction of the parent plant's stored energy (*MATING_ENERGY*) is given to the offspring.
  2. Mutualistic dispersal occurs for mutualistic plants. This action is carried out by the animal partner, which means that the 'seed' for a new individual can travel farther before it settles, and is done in the 'feeding' phase (see 5). The spatial extent of this dispersal event will depend on (1) the movements of the animal disperser after it has visited a mutualistic plant partner, (2) its efficiency in dispersing the 'seed' (*MUT_EFFICIENCY*), and (3) a cooling effect that decreases the dispersal (mutualistic) efficiency as time lapses (*MUT_COOLING*) (see Supplementary Table 1 for parameters governing this process and their explanation).The 'seed' of the parent plant is carried by the animal partner, so it may be placed beyond the distance-3 neighborhood. The choice of three cells of distance for offspring is arbitrary, but based on the fact that offspring of many species tend to be close to their parents range during the early stages of life (they can move afterwards, and this is considered in the model). Sensitivity analysis confirms that using other values yields similar results. If, before this time lapses (when the dispersal efficiency becomes zero), the animal partner comes across an empty cell in the landscape, it 'creates' an offspring for the plant previously visited with a probability given by its *MUT_EFFICIENCY*. Although plants can reproduce sexually, we only needed to simulate the ecological fact that plants can reproduce without the need of physically encountering another individual of the same species.

4) Movement

If the individual is a plant it does not move. Otherwise a neighboring cell (distance 1) is selected uniformly at random. If the selected cell contains a prey species, feeding occurs (see 5). Otherwise, if there is an available space in the selected cell, the individual moves there. The motion is therefore a two-dimensional random walk, as represented in Supplementary Figure 1.

5) Feeding

Having selected (in 4) to move into a cell containing prey, there are three possible trophic interactions:

1. Predation: If neither individual belongs to a basal species a predation event occurs with probability *CAPTURE_PROB*. The prey species dies and a fraction of its energy *EFFICIENCY_TRANS* is given to the predator. The predator moves into the new cell.

2. Herbivory: If one individual is a non-mutualistic animal, the other is a plant, and there is space to move into the selected cell, they interact. A fraction of the plant's energy *HERB_FRACTION* is lost, and a fraction (*HERB_EFFICIENCY*) of this energy is given to the herbivore. Both individuals continue living and the herbivore moves into the new cell. If the animal is an omnivore an additional trade-off (*OMNI_TRADEOFF*) is applied to its energy gained, since omnivore species are less efficient at digesting plant matter than straight herbivores.

3. Mutualism: If the individuals share a mutualistic link, and there is space for the animal to move, they interact. A fraction of the plant's energy (*MUT_FRACTION*) is transferred to the animal. The animal also keeps track of which plant it interacted with. If it later reaches an available cell in the landscape it creates a new individual belonging to this plant species, with probability MUT_EFFICIENCY. Seed release by herbivores is a decaying function of time and depends on a given probability. On each time step that an offspring is not produced, the mutualistic efficiency is reduced by a fraction *MUT_COOLING*.

6) Metabolic loss

If the individual is an animal it reduces its stored energy by a fraction *LIVING_EXPEND*, to account for metabolic losses. If the individual is a plant it auto-trophically increases its energy by a fraction *SYNTHESIS_ABILITY*. This, along with the randomly generated immigrants, are the only energy input to the system.

1.2 Model Parameters

During model development[1] a set of parameter values were selected that produced realistic community patterns and stable dynamics. In particular, the rank-abundance and degree-distributions were shown to be well fitted by log-normal and exponential functions, which is a quantitative pattern that has been observed in natural communitie3. Where possible, these parameters are based on ecological realism; the main example being trophic assimilation efficiency. It is well known that energy is lost when transferred between trophic levels, and that transfer rates are different depending on the type of resource consumed (plant vs. animal biomass)[2]. As such the assimilation rate is higher for plant biomass than animal biomass (*HERB_EFFICIENCY* > *EFFICIENCY_TRANS*). The extra reduction in transfer efficiency *OMNI_TRADEOFF* models the fact that omnivores are less well adapted to consume plant material because they also consume meat. Other than the omnivory trade-off all species within a functional group have identical parameters, and therefore differences between species are defined only by feeding relationships.

A key mechanism, and novel feature of the model, is mutualism. Mutualistic interactions are trophic, so energy is transferred from plant to consumer, but less than in an herbivorous interaction (*MUT_FRACTION* < *HERB_FRACTION* × *HERB_EFFICIENCY*). Therefore, a mutualistic animal benefits energetically from the interaction, but less so than if it were herbivorous. A mutualistic plant benefits significantly by having less of its resource consumed, and receiving improved dispersal ability. There is a potential disadvantage to the plant that it must wait for a partner to reproduce. However, the combined effect is that mutualism shifts some of the benefit of interaction in favor of the plant, whereas herbivory only benefits the consumer and harms the plant.

Lurgi et al. (2016)[1] conducted a sensitivity analysis, which showed that their results were not significantly affected by a ±10% variation in the value of all parameters (see S.I. in[1]). We conducted a sensitivity analysis for the results presented in the main text (see Supplementary Note 3). The default parameter values are given in Supplementary Table 1.

2 Modelling habitat loss

In order to study the effect of habitat loss (HL) on simulated communities we extend the IBM of Lurgi et al. (2016)[1] by implementing two HL algorithms (plus an additional one representing intermediate spatial correlation of HL). Simulations are set up and run as detailed in the previous sections but on the 1000th time step, after the initial transient dynamics, a given fraction of the lattice cells are destroyed simultaneously. The individuals inhabiting the destroyed cells are removed. Subsequently an individual may select a destroyed cell to move into (see section 1.1, above), in which case it is unable to move and remains in place. At each % of HL, we let disturbed communities evolve and pass a transient phase (4000 time steps), after which community metrics are calculated at the end of the simulation run. In the reproduction phase destroyed cells are counted as unavailable for the placement of offspring. Results are presented for incrementally affected landscapes, representing a gradient of habitat loss. The levels of destruction are referred to by the percentage of destroyed cells: HL = [0, 10, 20, ..., 90] %. The cells to destroy are chosen by two simple algorithms, giving two habitat loss scenarios: 1) Random and 2) Contiguous. These scenarios represent two extremes of the spatial pattern in which we may expect habitat to be destroyed in nature. Additionally, to explore the transition between both scenarios, we include a third algorithm that represents (3) Intermediate HL. This third scenario corresponds to a degree of spatial correlation (Moran's I) of approximately 0.5 (halfway between random and contiguous loss).

1) **Random habitat loss** proceeds by selecting lattice cells uniformly at random from the set of non-destroyed cells. This is repeated until the desired percentage HL is achieved. The result is a patchy and fragmented landscape.
2) **Contiguous habitat loss** proceeds by selecting a 'seed cell' uniformly at random from the pristine landscape. Destruction then spreads radially outwards from the seed cell, according to the distance metric defined in section 1 and the boundary conditions of the lattice that are used to avoid edge effects during the simulations. This results in contiguous regions of destroyed and pristine habitat.
3) **Intermediate habitat loss** proceeds by selecting a 'seed cell' uniformly at random from the pristine landscape. Destruction then spreads radially outwards from the seed cell. After each individual cell is destroyed the algorithm may switch, according to a set probability, to a new part of the landscape by randomly selecting a new seed cell. In the case of switching, habitat destruction then proceeds radially from the new seed cell. This process results in a patchy landscape with contiguous regions whose average size depends on the switching probability (P_{switch}). For P_{switch}=0 the algorithm is analogous to contiguous HL. For P_{switch}=1 it is analogous to random HL. In order to achieve an intermediate scenario between random and contiguous we selected P_{switch}=0.15 which produces landscapes with an average Moran's I of 0.5.

Supplementary Table 1. Definitions of model parameters, and default values used.

Parameter name	Value	Description
OCCUPIED_CELLS	0.4	Fraction of the grid initially occupied by individuals randomly placed on it.
MAX_RESOURCE	20	Maximum amount of resource an individual may possess at any given time.
MIN_RESOURCE	3	Death threshold: minimum amount of resource at individual may possess. Any individual possessing less than this amount at any given iteration will die (see text).
LIVING_EXPEND	0.01	Fraction of resource an individual spends in living every iteration of the model. Metabolic rate.
MATING_RESOURCE	0.5	Fraction of *MAX_RESOURCE* that is required for an individual to be able to reproduce.
MATING_ENERGY	0.2	Fraction of resource given to the offspring by the parent during reproduction. Each parent gives the same fraction. The total amount depends on how much resource the parent possesses at the time of reproduction.
IMMIGRATION	0.005	Probability that a new individual will appear in a cell of the grid each iteration. The species this individual belongs to is randomly chosen from the original species pool.
SYNTHESIS_ABILITY	0.1	Fraction of resource that is auto-trophically created by each individual from the basal species every iteration. This is the only energy input to the system.
HERB_FRACTION	0.7	Fraction of resource lost to herbivores by individuals belonging to a basal species during a trophic event, i.e. a species in the first trophic level feeding on a species in the basal level.
OMNI_TRADEOFF	0.4	Fraction of resource that omnivores are effectively able to gather when feeding on a species from the basal level (a plant).
MUT_FRACTION	0.25	Fraction of resource of a primary producer (basal species individual) that a mutualistic partner obtains when an interaction of this type occurs.
CAPTURE_PROB	0.4	Probability that a predator individual embarks upon a trophic relationship with one of its prey individuals when it encounters it.
EFFICIENCY_TRANS	0.2	Fraction of the resource the prey that is assimilated by the predator in a carnivorous interaction, i.e. trophic interaction not involving individuals from the basal species.
HERB_EFFICIENCY	0.8	Fraction of the resource of the prey assimilated by the herbivore in an herbivorous interaction.
MUT_EFFICIENCY	0.8	Efficiency of an individual mutualist when dispersing a plant partner. In other words, the probability with which a mutualistic individual will facilitate the creation of a new individual of the last species of plant it visited when it is positioned on an empty cell immediately after it interacted with a mutualistic plant partner.
MUT_COOLING	0.9	Cooling factor for the mutualistic efficiency of plant dispersers (mutualists). This is the fraction of mutualistic efficiency that remains after each iteration.
REPROD_RATE	0.01	Reproduction rate of non-mutualistic plant species. Probability with which an individual belonging to a plant species that does not possess mutualistic partners for dispersal will create an offspring in any given iteration of the simulation run.

Supplementary Note 2. Details of background and methodology for structural equation modeling, and additional results not included in the main text.

Structural equation models (SEMs) are increasingly used in ecology[4,5]. They are a type of path analysis, used for testing hypothesised causal relationships between multiple variables. Each path in the SEM represents a hypothesised (directed) causal relationship between two variables. Data is collected for each variable and multivariate models fitted. A goodness of fit test is then used to try to refute the hypothesised causal structure based on the observed data. Classical SEMs make the assumptions that all variables follow a multivariate normal distribution, and that all observations are independent. Piecewise SEMs relax these assumptions and introduce more flexibility into the modelling framework[6].

Supplementary Figure 2. Example of a hypothesised causal structure between four variables (A, B, C and D).

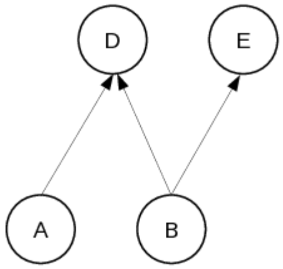

The goodness of fit of piecewise SEMs is tested using the directed separation (*d-sep*) test proposed by[4]. The d-sep test is derived from graph theoretic methods for analysis of directed-acyclic graphs. In the SEM context, it is a test of the conditional independence claims implied by the model structure. This is clearest to see by means of an example. Supplementary Figure 2 shows an example SEM structure for four variables. The depicted SEM implies that variables D and E are conditionally independent given A and B. The evidence for this relationship would be tested using the models *D~f(A,B)* and *E~f(B)*. If there is insufficient evidence to reject any of the conditional independence claims implied by the hypothesised structure, then the data is said to support this model of causality. Furthermore, the coefficients of the models fitted to the data can tell us about the relationships between variables (i.e. the size and sign of the causal effect of one variable on another). The Akaike information criterion (AIC) may be used to select for the causal model that best fits the data[4].

In order to simplify the SEMs, we selected a subset of variables that we feel captures the most important community responses. In particular, we omitted those variables which displayed no significant trends in either HL scenario (compartmentalisation, nestedness, number of species), those which changed significantly in one HL scenario but not the other (generality, vulnerability, Shannon metrics), and those that changed exactly in the same way (abundance decreased similarly across HL scenarios), thereby allowing direct comparison between the SEMs random and contiguous HL. We selected CV as the main output variable for the modelling. As such the SEMs can be thought of as an evaluation of the various mechanisms driving changes in stability under HL.

The above simplifications result in six variables, including HL (the level of HL), which we use for the structural equation modelling. Based on evidence taken from the literature we hypothesise a set of causal relationships between the variables. These relationships and the arguments for their existence are given in Supplementary Table 2. Together this set of relationships results in the hypothesised causal structure depicted in Supplementary Figure 3, which we test using the data.

Supplementary Figure 3. SEM model structure.

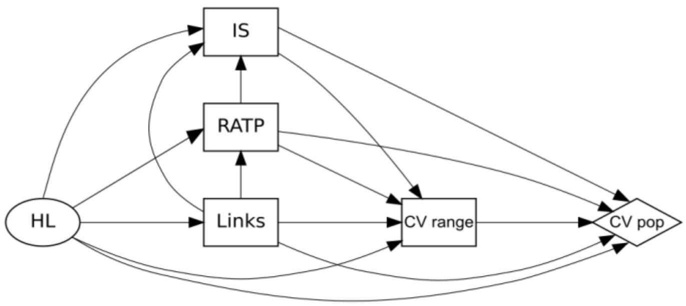

The hypothesised causal pathways of mechanisms driving changes in stability under HL. Each arrow represents a causal link. Variables are as defined in the text.

SEMs were fitted for each fraction of mutualism independently (the results are summarised in Supplementary Table 3). Since the fraction of mutualism made little qualitative difference in the SEM results (did not change the sign of significant links except in a handful of cases), we decided to aggregate over fractions of mutualism. This aggregation effectively increased the number of replicate simulations at each level of HL. The aggregate results for all fractions of mutualism are those presented in the main text, and are also summarised in Supplementary Table 3. The one response for which the trend directions was sensitive to the fraction of mutualism was CV range. In a previous work, communities with high fractions of mutualism were found to display greater spatial aggregation[1] compared to communities with lower fractions of mutualism. It may be that this difference in spatial organisation is involved in subtly mediating community responses to HL. However, this does not affect our interpretation of the results as presented in the main text.

We implemented SEM analysis using the R package *PiecewiseSEM*, using linear models to model the relationships between variables. Prior to fitting the models, variables were transformed to ensure linearity and normality. Specifically, all variables, except for HL, were standardised. Additionally, IS and CV were log-transformed prior to standardising, while RATP was square root transformed. We then fitted the fully-connected SEM model (Supplementary Figure 3) and, following Shipley's methodology[4], iteratively removed the least significant links (the linear model with the highest F-test p-value) to obtain the simplest causal model consistent with the data. On each iteration, the SEM was refitted and one link removed, and the AIC_c and Fisher's p-value tested. The iterative link removal was terminated when either 1) the change in AIC_c from the baseline model was greater than 3, or 2) there was evidence to reject the conditional independence claims at 95% confidence (Fisher's p-value < 0.05). The best SEM was taken as the simplest model structure achieved prior to termination. The coefficients of this SEM (scaled to allow inter-model comparison) were taken as quantifying the causal effect of one variable on another.

Supplementary Table 2. Hypothesised causal links between the variables selected for the structural equation modelling.

Predictor	Response	Argument for causal link
HL	Links	Loss of links resulting directly from HL. Various mechanisms could drive such a change: fewer individuals, increased competition for resources, reduced dispersal[8,9].
HL	RATP	Disproportionate impact of top predators relative to other trophic levels is well established impact of HL[7,10,11], perhaps due to reduced productivity of the habitat cascading through food-chains.
HL	IS	Based on the mobility experiment (main text, figure 4) we proposed there is a direct effect of HL on IS (that differs between HL scenarios). Some empirical evidence that HL can alter interaction strengths is offered by[12,13].
HL	CV population and CV range	There is a direct causal impact of HL on the stability response that is not mediated through any other variable.
Links	RATP	Relative abundance of top predators, the argument being that more links in the network represent more trophic pathways for energy transfer to the top level[14].
Links	IS	A changing number of links represents a change in network topology, which can affect the mean interaction strength (via the distribution of interaction strengths). In particular, the loss of links is likely to be associated with links getting weaker, which tends to reduce the mean IS.
Links	CV population and CV range	The classic argument following May's seminal work[15] is that high connectance is bad for asymptotic stability. However, such a relationship has been demonstrated to be topology dependent[16]. Nevertheless, we argue that increasing the number of links is likely to increase both abundance and range area variability.
RATP	IS	Changes in relative trophic abundances will impact the interaction strength distribution. We argue that a shift towards basal species presents a loss of predation and therefore a reduction in interaction strengths, although this may change if the food webs display non-random and non-homogeneous distributions of strong and weak link1[8].
RATP	CV population and CV range	There is some evidence that predation drives increased temporal variability[17]. We argue that a shift towards basal species will reduce temporal stability in abundances and in range area.
IS	CV population	Theoretical argument1[5,19] and empirical evidence[20] that strong trophic interactions produce high temporal variability. Additionally, strong correlation as shown in figure 2 of the main text.
IS	CV range	Hypothesised based on the above (nothing in the literature that we are aware of) if increased IS drives increase in abundance variability, perhaps it also drives increase in range area variability (main text, figure 1).
CV range	CV population	If, on average, species range areas become more variable this is likely to drive increased abundance variability. However, the types of variability are distinct. It is possible for CV range to increase without CV population (and vice versa).

Supplementary Table 3. Summary results of SEMs for all individual fractions of mutualism (FM), compared to those of for the aggregate data (ALL_FM) presented in the main text.

CONTIGUOUS HABITAT LOSS

Response	Predictor	FM_0	FM_0.1	FM_0.2	FM_0.3	FM_0.4	FM_0.5	FM_0.6	FM_0.7	FM_0.8	FM_0.9	FM_1	Mean	Variance	All_FM
Links	HL	-0.135		-0.116	-0.067	-0.085		-0.193	-0.150	-0.181	-0.164	-0.201	-0.144	0.002	-0.107
RATP	Links	0.230	0.239	0.399	0.346	0.401	0.437	0.391	0.460	0.526	0.371	0.663	0.406	0.015	0.542
RATP	HL	-0.080										0.089	0.004	0.014	
IS	HL	0.668	0.542	0.534	0.555	0.566	0.513	0.458	0.549	0.546	0.554	0.552	0.549	0.002	0.435
IS	Links	0.288	0.197	0.289	0.263	0.210	0.178	0.170	0.240	0.179	0.173	0.159	0.213	0.002	0.207
IS	RATP		0.194	0.231	0.344	0.341	0.329	0.332	0.334	0.397	0.420	0.400	0.332	0.005	0.298
CV range	HL	0.506	0.599	0.430	0.403	0.437	0.369	0.533	0.382	0.263	0.383	0.218	0.411	0.012	0.386
CV range	Links	0.212	0.409	0.241	0.421	0.366	0.285	0.346	0.377	0.261	0.360	0.293	0.325	0.005	0.363
CV range	RATP	0.092		0.122	0.088	0.129	0.165	0.311	0.207	0.156	0.304	0.236	0.181	0.007	0.190
CV range	IS		-0.384					-0.352					-0.368	0.000	-0.151
CV pop	IS	0.936	0.774	0.708	0.789	0.828	0.689	0.944	0.967	0.919	0.876	1.013	0.859	0.012	0.878
CV pop	CV range	0.311	0.201	0.280	0.255	0.289	0.296	0.260	0.348	0.296	0.363	0.402	0.300	0.003	0.295
CV pop	HL	-0.263				-0.117			-0.201	-0.185	-0.138	-0.198	-0.184	0.003	-0.080
CV pop	Links	-0.173	-0.107		-0.106	-0.158	-0.100	-0.105	-0.219	-0.180	-0.251	-0.250	-0.165	0.004	-0.154
CV pop	RATP			-0.174	-0.125	-0.159	-0.080	-0.118	-0.200	-0.074	-0.126	-0.089	-0.127	0.002	-0.116

RANDOM HABITAT LOSS

Response	Predictor	FM_0	FM_0.1	FM_0.2	FM_0.3	FM_0.4	FM_0.5	FM_0.6	FM_0.7	FM_0.8	FM_0.9	FM_1	Mean	Variance	All_FM
Links	HL	-0.141	-0.111	-0.219	-0.083	-0.090	-0.084	-0.150	-0.139	-0.181	-0.166	-0.162	-0.139	0.002	-0.115
RATP	Links	0.182	0.157	0.187	0.195	0.159	0.233	0.204	0.140	0.283		0.243	0.198	0.002	0.266
RATP	HL	-0.524	-0.513	-0.463	-0.315	-0.367	-0.286	-0.383	-0.326	-0.224	-0.310	-0.200	-0.356	0.012	-0.318
IS	HL	-0.590	-0.527	-0.456	-0.215	-0.161	-0.129	-0.064	-0.049				-0.274	0.047	-0.116
IS	Links	0.437	0.483	0.369	0.301	0.397	0.290	0.266	0.273	0.366	0.245	0.250	0.334	0.007	0.296
IS	RATP	0.104	0.228	0.347	0.561	0.516	0.572	0.444	0.557	0.590	0.668	0.699	0.481	0.034	0.441
CV range	HL	0.819	0.240	0.228					-0.128	-0.122	-0.157	-0.193	0.098	0.134	-0.048
CV range	Links	-0.697	-0.391	-0.413		-0.283	-0.320	-0.111	-0.268	-0.304	-0.337	-0.301	-0.343	0.022	-0.189
CV range	RATP	0.132	-0.135				-0.118					-0.204	-0.081	0.022	-0.067
CV range	IS	1.564	0.776	0.766	0.354	0.568	0.641	0.566	0.627	0.451	0.538	0.810	0.696	0.102	0.436
CV pop	IS	0.132			0.106		0.246	0.236	0.122	0.170	0.210	0.140	0.170	0.003	0.145
CV pop	CV range	0.819	0.816	0.711	0.731	0.646	0.708	0.725	0.594	0.971	0.676	0.450	0.713	0.018	0.705
CV pop	HL	-0.222	-0.286	-0.271	-0.184	-0.202	-0.224	-0.165	-0.164	-0.142	-0.179	-0.106	-0.195	0.003	-0.111
CV pop	Links						-0.079				-0.074		-0.076	0.000	-0.029
CV pop	RATP				0.076								0.076		

Best fit model shown in each case. Values given are standardised model coefficients, which quantify effect sizes. Non-significant links are not shown. Coefficients which differ in sign from the aggregate data model are highlighted in yellow.

Supplementary Note 3. Sensitivity analysis.

We investigated the sensitivity of our SEM results to variation in the model parameters. We used a latin hypercube sampling[21] to explore a region of parameter space. The latin hypercube is a sampling method that aims to produce a random distribution of points which sample a high dimensional space evenly and efficiently. Such a sampling of 20 points is illustrated in Supplementary Figure 4, for a two-dimensional slice of parameter space (with ±10% variation in parameter value). It generalises trivially to higher dimensions. Using this method, we drew 400 samples from a region of the full 17-dimensional parameter space, defined by ±20% of the original parameter values (as given in Supplementary Note 1). Each of these 400 samples represents a unique parametrisation of the model. We then ran model simulations for each parametrisation at all 10 values of habitat loss (0-90%) for three of the fractions of mutualism (0.0, 0.5, 1.0). The use of only these three fractions of mutualism (instead of all 11 used previously) reduced the number of simulations that needed to be run, while retaining a representative spread between mutualism and antagonism. The set of simulations run with these randomly selected parameter values are referred to below and in the main text as the sensitivity ensemble.

Supplementary Figure 4. Example of a latin hypercube sample.

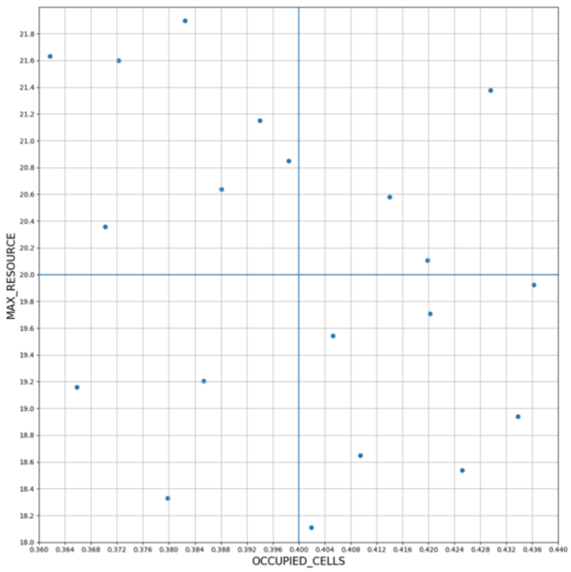

The example shows 20 data points drawn from a 2-dimensional slice of parameters. Space defined by ±20% of the original parameter values (blue lines). The procedure generalises trivially to larger samples and more dimensions.

We then fitted a structural equation model to the data generated by the sensitivity ensemble simulations. This SEM modelling followed the same procedure detailed in Supplementary Note 2. The SEM results for the sensitivity ensemble are compared to those for the original parameters in Supplementary Table 4. It is clear from this table that the parameter randomisation has little qualitative effect on the structure of the SEMs, since the signs of the significant links are not altered. There are a number of cases where the links are significant under the sensitivity ensemble but not in the original results. This is to be expected given that the sensitivity ensemble contains more replicate simulations at each value of HL.

Supplementary Table 4. Sensitivity of SEM results to ±20% variation in parameter values.

Contiguous Habitat Loss

Response	Predictor	AGGREGATE FM		FM = 0.0		FM = 0.5		FM = 1.0	
		Original Parameters	Sensitivity Ensemble	Original Parameters	Sensitivity Ensemble	Original Parameters	Sensitivity Ensemble	Original Parameters	Sensitivity Ensemble
Links	HL	-0.107	-0.069	-0.135	-0.052		-0.069	-0.201	-0.116
RATP	Links	0.542	0.686	0.230	0.157	0.437	0.645	0.663	0.734
RATP	HL			-0.080	-0.049			0.089	0.041
IS	HL	0.435	0.414	0.668	0.520	0.513	0.431	0.552	0.460
IS	Links	0.207	0.258	0.288	0.272	0.178	0.253	0.159	0.194
IS	RATP	0.298	0.259		0.067	0.329	0.249	0.400	0.347
CV range	HL	0.386	0.334	0.506	0.519	0.369	0.380	0.218	0.250
CV range	Links	0.363	0.414	0.212	0.304	0.285	0.437	0.293	0.391
CV range	RATP	0.190	0.193	0.092	0.136	0.165	0.175	0.236	0.238
CV range	IS	-0.151	-0.219		-0.273		-0.293		-0.146
CV pop	IS	0.878	0.594	0.936	0.749	0.689	0.623	1.013	0.686
CV pop	CV range	0.295	0.346	0.311	0.319	0.296	0.343	0.402	0.475
CV pop	HL	-0.080	-0.040	-0.263	-0.167		-0.024	-0.198	-0.069
CV pop	Links	-0.154	-0.301	-0.173	-0.260	-0.100	-0.302	-0.250	-0.394
CV pop	RATP	-0.116	-0.033		0.085	-0.080	-0.053	-0.089	-0.035

Random Habitat Loss

Response	Predictor	AGGREGATE FM		FM = 0.0		FM = 0.5		FM = 1.0	
		Original Parameters	Sensitivity Ensemble	Original Parameters	Sensitivity Ensemble	Original Parameters	Sensitivity Ensemble	Original Parameters	Sensitivity Ensemble
Links	HL	-0.115	-0.078	-0.141	-0.094	-0.084	-0.081	-0.162	-0.090
RATP	Links	0.266	0.448	0.182	0.192	0.233	0.361	0.243	0.475
RATP	HL	-0.318	-0.271	-0.524	-0.413	-0.286	-0.298	-0.200	-0.166
IS	HL	-0.116	-0.137	-0.590	-0.396	-0.129	-0.134		-0.032
IS	Links	0.296	0.318	0.437	0.370	0.290	0.312	0.250	0.325
IS	RATP	0.441	0.300	0.104	0.070	0.572	0.306	0.699	0.480
CV range	HL	-0.048	-0.047	0.819	0.374		-0.059	-0.193	-0.109
CV range	Links	-0.189	-0.239	-0.697	-0.411	-0.320	-0.235	-0.301	-0.337
CV range	RATP	-0.067		0.132	0.179	-0.118		-0.204	-0.030
CV range	IS	0.436	0.398	1.564	1.044	0.641	0.432	0.810	0.439
CV pop	IS	0.145	0.105	0.132	0.090	0.246	0.123	0.140	0.122
CV pop	CV range	0.705	0.629	0.819	0.584	0.708	0.631	0.450	0.774
CV pop	HL	-0.111	-0.078	-0.222	-0.077	-0.224	-0.113	-0.106	-0.102
CV pop	Links	-0.029	-0.040		-0.036	-0.079	-0.052		-0.050
CV pop	RATP		0.022						0.039

Standardised SEM coefficients compared between sensitivity ensemble and original (default parameter) simulations, for three fractions of mutualism (0.0, 0.5, 1.0).

Supplementary Note 4. Effects of varying immigration rate on species extinctions, interaction strength and stability.

To investigate the sensitivity of our results to changes in the rate of immigration, we conducted additional simulations for random and contiguous habitat loss (HL). The value of HL is varied between 0% and 90% in steps of 10%, as before. At each value of HL, we run replicates at 10 different immigration rates (IR):

IR = $\{1x10^{-4}, 2x10^{-4}, 3x10^{-4}, 4x10^{-4}, 5x10^{-4}, 1x10^{-3}, 2x10^{-3}, 3x10^{-3}, 4x10^{-3}, 5x10^{-3}\}$

These simulations thus explore a two-dimensional section of the parameter space, defined by the axes HL and IR. Simulations are run for three fractions of mutualism (0.0, 0.5, 1.0), giving the full range between antagonism (trophic) and mutualism. We focus on the effects that varying IR has on key aspects of our species interaction networks, that is, the number of extinctions, the strength of species interactions and the stability – temporal variability in population abundances – of the communities, which is the key result of our model when IR is high and no extinctions occur.

Results are presented as heat-maps over parameter space. Each pixel in the heat-map corresponds to a unique pair of HL and IR values, with the color given by the corresponding mean value of the variable studied in question (averaged over 25 replicates). In this way, it is possible to gain a qualitative impression of how the studied variables respond as HL and IR are varied.

Effects of varying IR on species extinctions

The number of extinctions increases as IR is reduced (Supplementary Figure 5). On average communities with higher fractions of mutualisms exhibit more extinctions, and contiguous HL produces more extinctions than random HL. In the contiguous scenario, the number of extinctions increases along the HL gradient, whereas this trend is less clear in the random scenario. Under random HL the dependence of extinctions on the level of HL appears to be reduced, especially for more mutualistic communities. In agreement with the results from the main text, no extinctions are reported at high IR.

Reducing IR increases the number of extinctions due to a weaker rescue effect. We find that more extinctions are produced by contiguous than by random HL, and that the number of extinctions under the random scenario is less sensitive to the level of HL. These observations suggest that the mechanism behind species extinctions differs between the two types of HL. Extinctions in the contiguous scenario might be due to strong predation driven by high IS, whereas in the random scenario they are likely due to changes in the network structure of the community due to low IS, although this is beyond the scope of this study. Results also suggest that the number of extinctions at low IR is slightly higher when the fraction of mutualism is high. However, given the small quantitative effect and the fact that the fraction of mutualism in our study refers to the proportion of mutualistic interactions in relation to herbivore links

in the second trophic level rather than to the whole set of interactions in the community (see main text), we cannot conclude that higher mutualism is detrimental for species persistence.

Supplementary Figure 5. Number of species extinctions at each combination of HL and IR

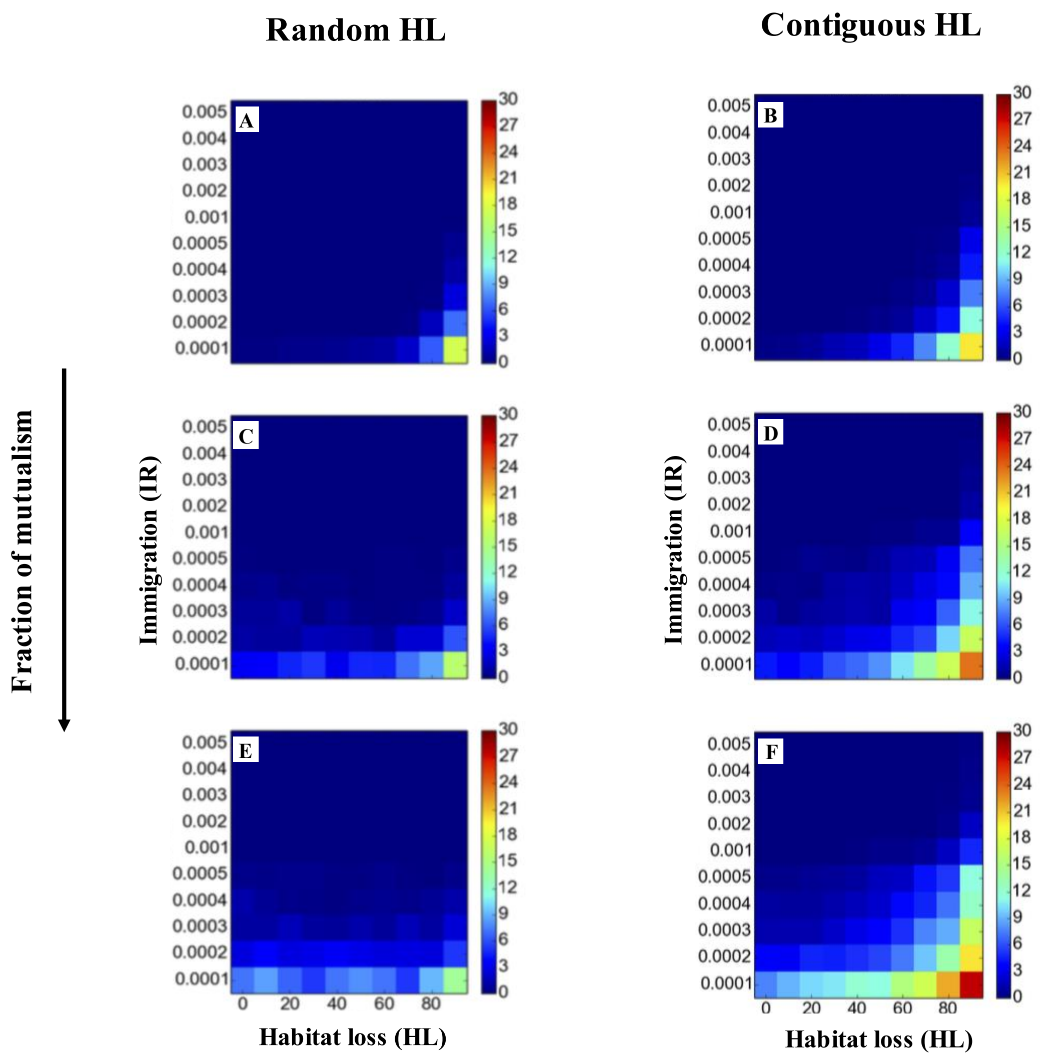

Each row corresponds to a different fraction of mutualism (0.0, 0.5, 1.0). The color scale represents the number of species going extinct.

Effects of varying IR on interaction strength and community stability

Contiguous HL increases both interaction strength and the temporal variability of population abundances (Supplementary Figure 6). Therefore, varying IR does not alter the direction of the response of these variables to contiguous HL that is reported in communities with high

IR. Reducing IR increases temporal variability and slightly increases the mean interaction strength, an effect that is more pronounced at lower fractions of mutualism.

The random scenario produces qualitatively the same patterns in the number of interactions as seen in the contiguous scenario (Supplementary Figure 7, panels B, E, H). However, random HL results in a slightly greater decline in interaction strength, in agreement with results representing high IR. As in the contiguous scenario, reducing IR increases temporal variability, with an associated increase in interaction strength (Supplementary Figure 7). However, the random scenario displays a subtler interaction between variability and IS. At all IR values, the gradient of increasing HL causes variability to first decrease, but then increase at extreme HL values. The role of IR is such that the net change in variability across the HL gradient shifts from a decrease (at high IR) to an increase (at low IR). This effect holds across all fractions of mutualism. Broadly, these changes in variability correlate with the changes in IS.

The results with varying IR are generally consistent with those of the main text, i.e. community responses to HL do not qualitatively change when IR is varied. The exception to this is that, at low IR, random HL results in a net increase in temporal variability of population abundances, rather than a net decrease. The increase in variability occurs at high levels of HL (> 70%), and is most visible at HL= 90% where the number of individuals is lowest. It is worth noting that CV population tends to infinity as the number of individuals tends to zero. This property of the metric may explain the apparent increase in variability in highly impacted landscapes at low IR. Collectively, we can conclude that, irrespective of IR, random HL reduces species interaction strengths, and therefore reduces temporal variability, whereas the converse holds for contiguous HL.

Supplementary Figure 6. Mean interaction strength (IS) and temporal variability of population abundances (CV Population) under **contiguous HL**.

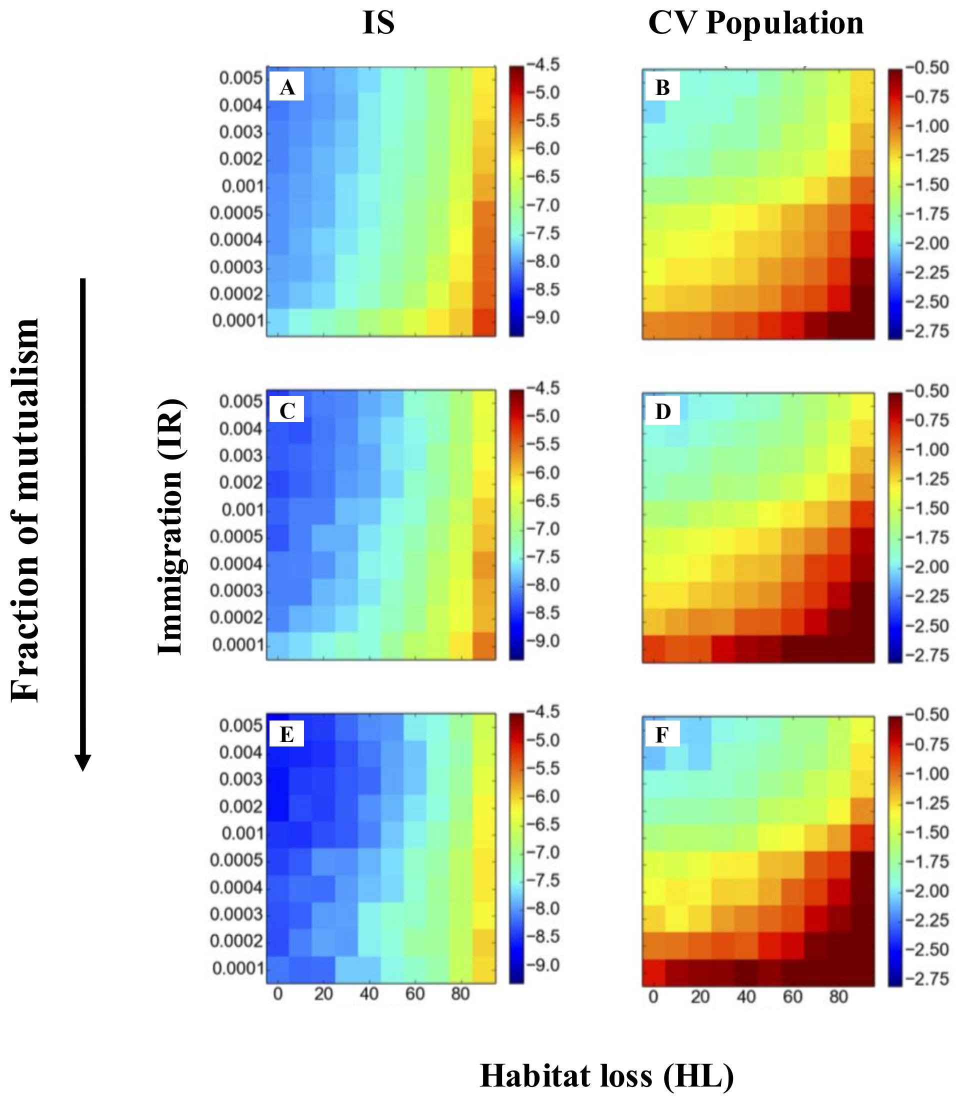

Each row corresponds to a different fraction of mutualism (0.0, 0.5, 1.0) (blue color = lower values; red color = higher values of the variable). Average values over 25 replicate simulations are shown.

Supplementary Figure 7. Mean interaction strength (IS) and temporal variability of population abundances (CV Population) under **random HL**.

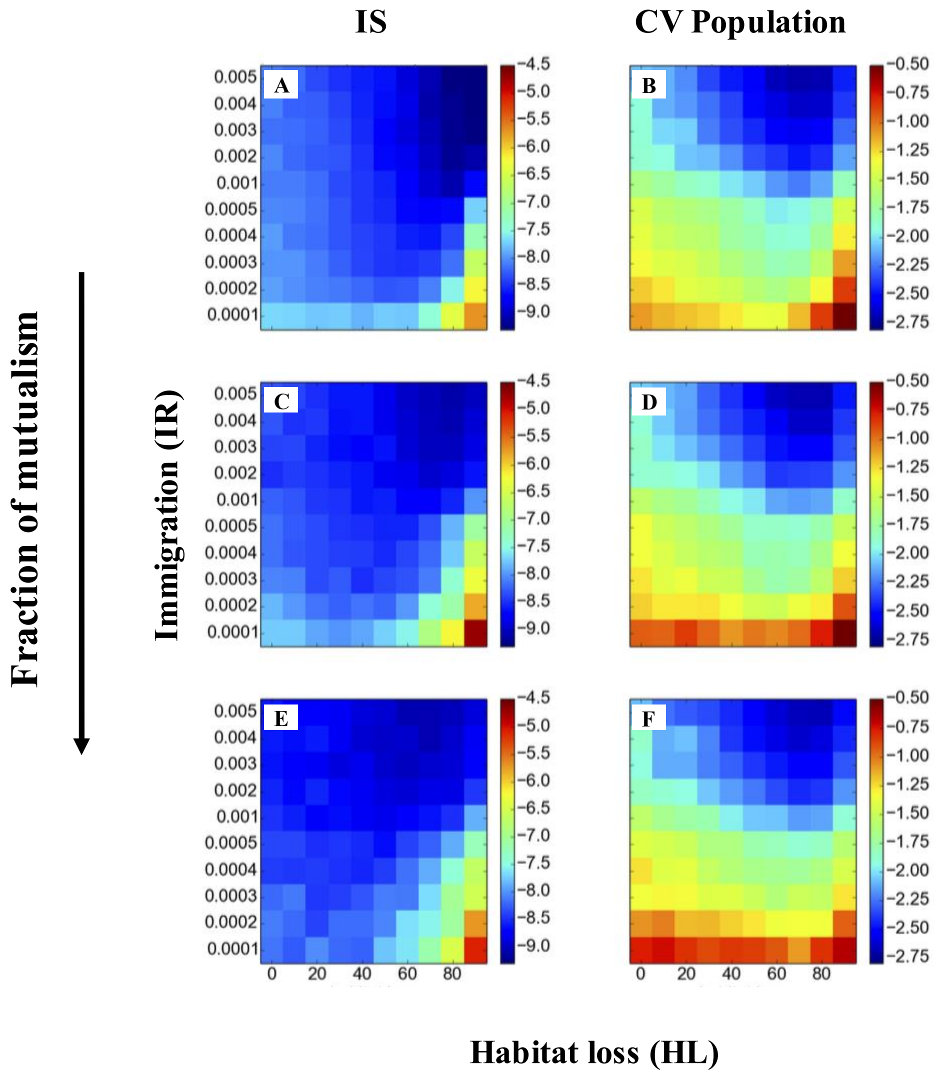

Each row corresponds to a different fraction of mutualism (0.0, 0.5, 1.0) (blue color = lower values of the variable; red color = higher values of the variable). Average values over 25 replicate simulations are shown.

Supplementary Figures

Supplementary Figure 8. Total number of individuals against percentage habitat loss for the random (left), intermediate (middle) and contiguous (right) scenarios.

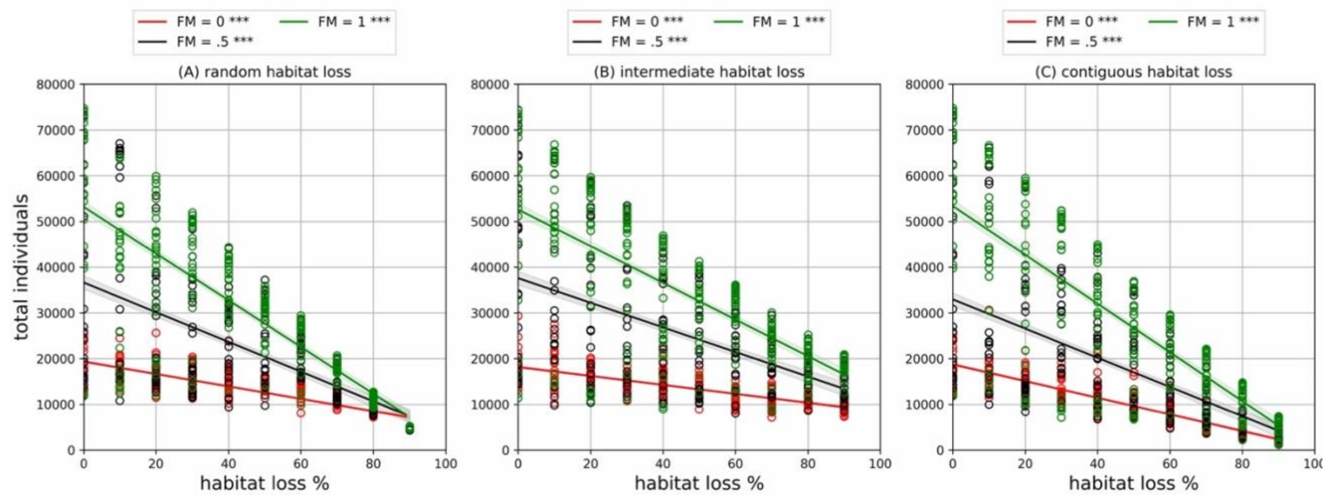

Three fractions of mutualism (FM) are shown, with linear models fitted. Marker in the legends indicate trend p-values as follows: ***, **, * and + for p <0.001, 0.05, 0.1 and 0.5 respectively (F-test). Circles represent single simulation runs. Shaded regions indicate the standard error of the mean.

Supplementary Figure 9. Nestedness (NODF) against percentage habitat loss for the random (left), intermediate (middle) and contiguous (right) scenarios.

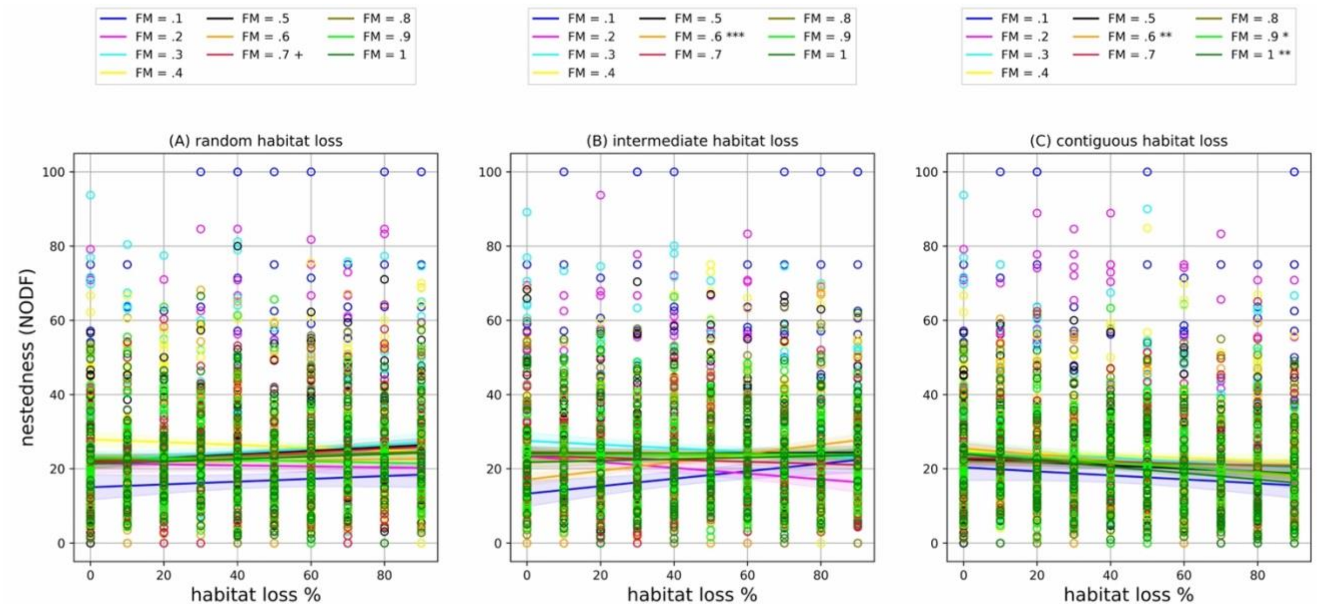

All eleven fractions of mutualism (FM) are shown, with linear models fitted. Marker in the legends indicate trend p-values as follows: ***, **, * and + for p <0.001, 0.05, 0.1 and 0.5 respectively (F-test). Circles represent single simulation runs. Shaded regions indicate the standard error of the mean.

Supplementary Figure 10. Similar to figure 1, but for relative abundance of top predator populations (RATP). Here only three fractions of mutualism (FM) are shown, for clarity.

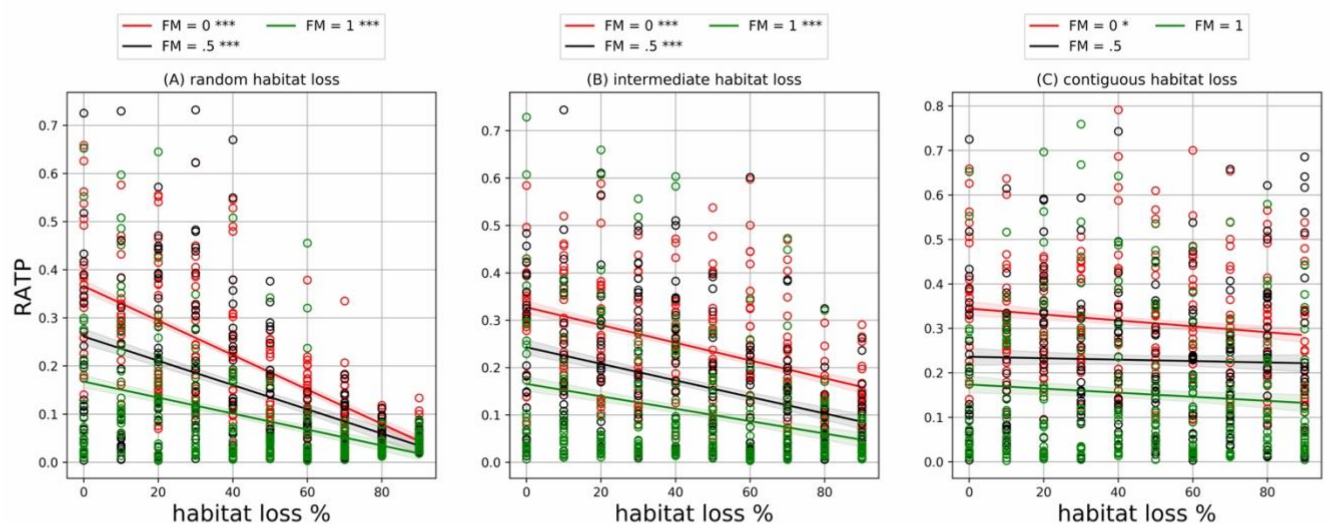

Supplementary Figure 11. Similar to figure 1, but for CV range. Here only three fractions of mutualism (FM) are displayed, for clarity.

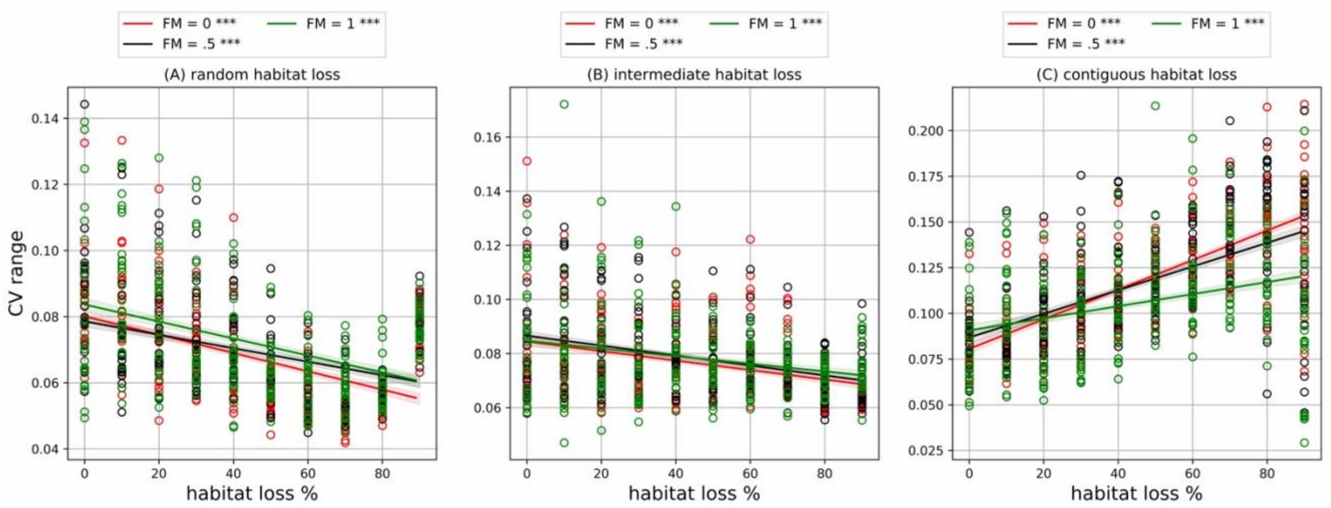

Supplementary Figure 12. Interaction strength distributions as a function of habitat loss. For clarity, values are aggregated over all replicated communities at two fractions of mutualism (FM).

RANDOM

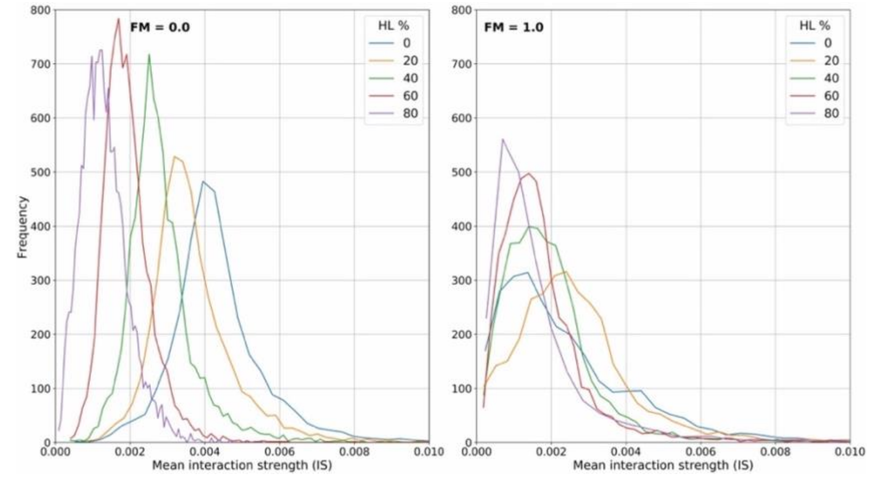

INTERMEDIATE

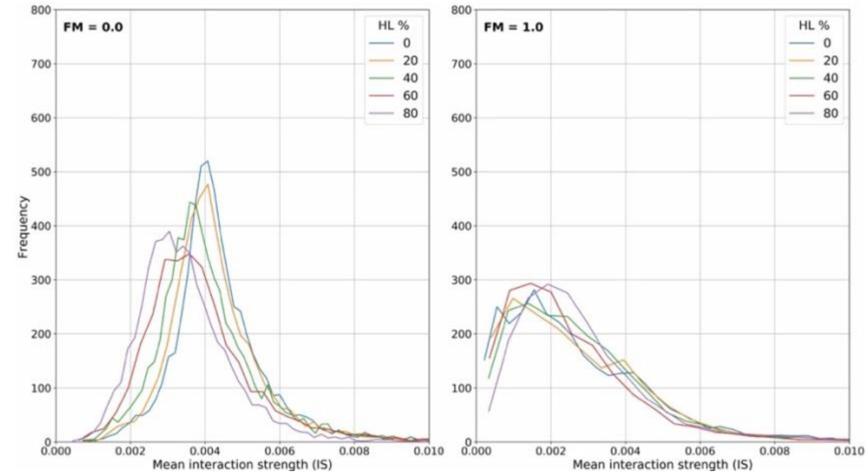

CONTIGUOUS

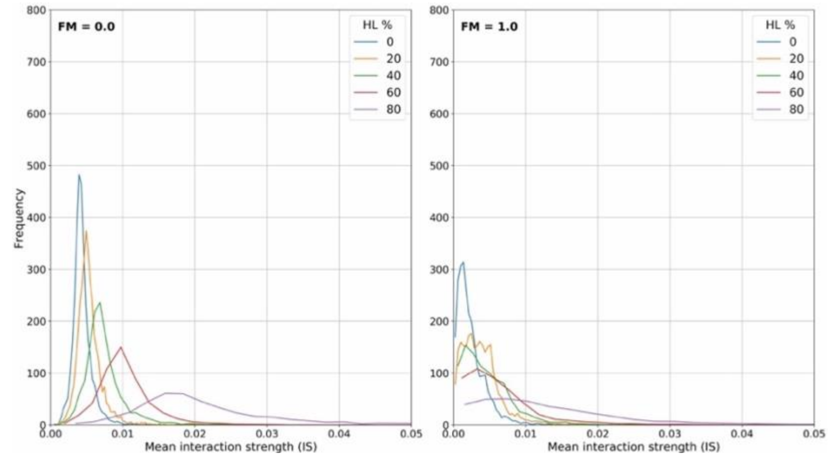

Supplementary Figure 13. Example of rank-abundance distributions for undisturbed communities (HL = 0%), for three fractions of mutualism (FM): (A) FM = 0%; (B) FM = 50%; (C) FM = 100%.

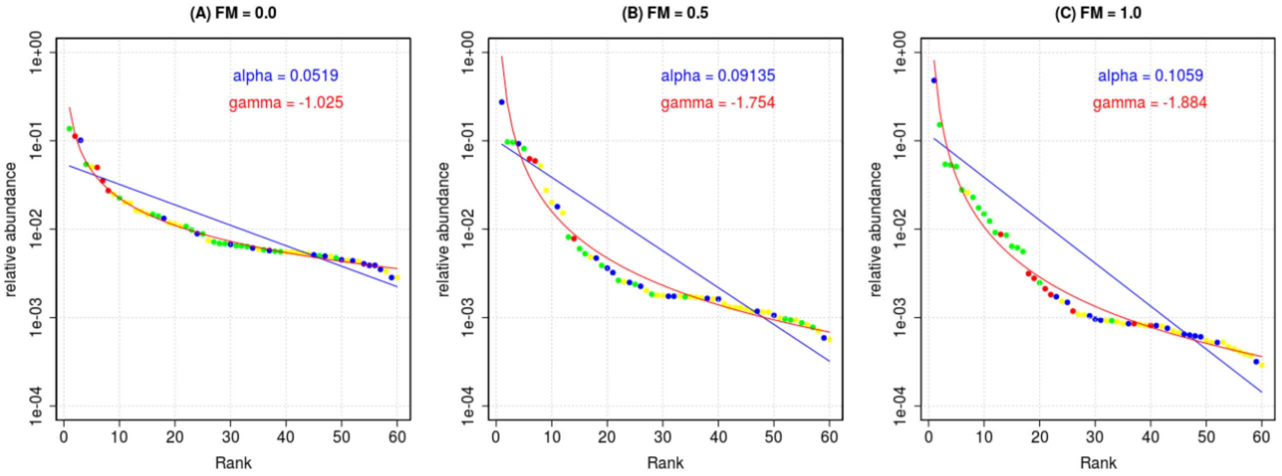

Species abundances are relative to the total number of individuals in the community, and plotted on a logarithmic scale. Circles represent species, coloured according to trophic level: green=basal, blue=herbivore/mutualist animal; yellow=primary predator; red=top predator. Blue and red lines give the pre-emption and Zipf model fits respectively, two types of log-normal distributions associated to fits rank-abundance in empirical communities. The best-fit parameter value for each model is provided in the plot.

Supplementary Figure 14. Example of cumulative degree distributions for undisturbed communities (HL = 0%), for three fractions of mutualism (FM): (A) FM = 0%; (B) FM = 50%; (C) FM = 100%.

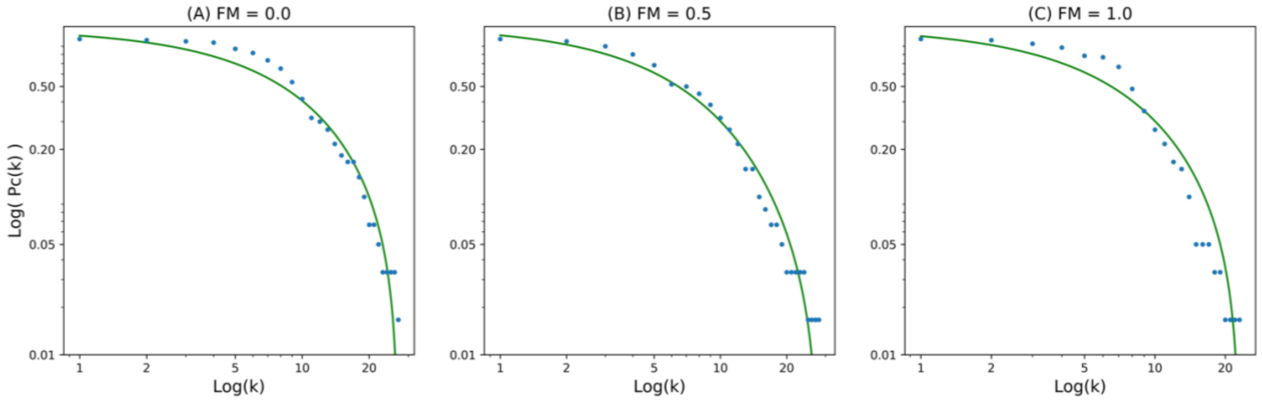

Lines represent a fit of each dataset to an exponential distribution (p values for all fits <0.001). Axes are in a logarithmic scale.

Supplementary References

1. Lurgi, M., Montoya, D. & Montoya, J.M. The effects of space and diversity of interaction types on the stability of complex ecological networks. *Theor. Ecol.* **9**, 3–13 (2016).
2. Ings, T.C., Montoya, J.M., Bascompte, J., Blüthgen, N., Brown, L. Dormann, C.F., et al. Review: Ecological networks–beyond food webs. *J. Anim. Ecol.* **78**, 253–269 (2009).
3. Montoya, J.M., Pimm, S.L. & Solé, R.V.. Ecological networks and their fragility. *Nature* **442**, 259–264 (2006).
4. Shipley, B. The AIC model selection method applied to path analytic models compared using ad-separation test. *Ecology* **94**, 560–564 (2013).
5. Yvon-Durocher, G. Allen, A. P. Cellamare, M. Dossena, M. Gaston, K. J. Leitao, M. et al. Five years of experimental warming increases the biodiversity and productivity of phytoplankton. *PLoS Biol.* **13**, e1002324 (2015).
6. Lefcheck, J. S. piecewiseSEM: Piecewise structural equation modelling in r for ecology, evolution, and systematics. *Methods Ecol. Evol.* **7**, 573-579 (2016).
7. Solé, R. V. & Montoya, J. M. Ecological network meltdown from habitat loss and fragmentation. *Ecological Networks: Linking Structure to Dynamics in Food Webs*, 305-323 (2006).
8. Brose, U., Ostling, A., Harrison, K. & Martinez, N.D. Unified spatial scaling of species and their trophic interactions. *Nature* **428**, 167-171 (2004).
9. Galiana, N., Lurgi, M., Claramunt-López, B., Fortin, M.-J., Lerouz, S., Cazelles, K. et al. The spatial scaling of species interaction networks. *Nature Ecol. Evol.* **2**, 782–790 (2018).
10. Duffy, J. E. Biodiversity loss, trophic skew and ecosystem functioning. *Ecol. Lett.* **6**, 680-687 (2003).
11. Raffaelli, D. How extinction patterns affect ecosystems. *Science* **306**, 1141-1142 (2004).
12. Hagen, M., Kissling, W.D., Rasmussen, C., De Aguiar, M.A., Brown, L.E., Carstensen, D. W. et al. Biodiversity, species interactions and ecological networks in a fragmented world. *Adv. Ecol. Res.* **46**, 89-210 (2012).
13. Tylianakis, J. M., Didham, R. K., Bascompte, J., & Wardle, D. A. Global change and species interactions in terrestrial ecosystems. *Ecol. let.* **11**, 1351-1363 (2008).
14. Turney, S., & Buddle, C. M. Pyramids of species richness: the determinants and distribution of species diversity across trophic levels. *Oikos* **125**, 1224-1232 (2016).
15. May, R. M. Will a large complex system be stable?. *Nature* **238**, 413-414 (1972).
16. McCann, K.S. The diversity–stability debate. *Nature* **405**, 228-233 (2000).
17. McCann, K.S., Rasmussen, J.B., & Umbanhowar, J. The dynamics of spatially coupled food webs. *Ecol. lett.* **8**, 513-523 (2005).
18. Neutel, A.M., Heesterbeek, J.A. & De Ruiter, P.C. Stability in real food-webs: weak links in long loops. *Science* **296**, 1120-1123 (2002).
19. Gross, T., Rudolf, L., Levin, S. A., & Dieckmann, U. Generalized models reveal stabilizing factors in food webs. *Science* **325**, 747-750 (2009).
20. O'Gorman, E. J., & Emmerson, M. C. Perturbations to trophic interactions and the stability of complex food webs. *P. Natl. Acad. Sci. USA* **106**, 13393-13398 (2009).
21. Helton, JC. Davis, FJ. Latin hypercube sampling and the propagation of uncertainty in analyses of complex systems. *Reliability Engineering & System Safety*, **81**(2003).
