# Supplementary material for: The stability of multitrophic communities under habitat loss
Source: Nat Commun. 2019 May 24;10:2322. doi: 10.1038/s41467-019-10370-2 (PMC6534601; doi:10.1038/s41467-019-10370-2)
Supplement: Supplementary file 3 — Reporting Summary [file 41467_2019_10370_MOESM3_ESM.pdf]

nature research

Corresponding author(s): Daniel Montoya

Last updated by author(s): Apr 10, 2019

Reporting Summary

Nature Research wishes to improve the reproducibility of the work that we publish. This form provides structure for consistency and transparency in reporting. For further information on Nature Research policies, see Authors & Referees and the Editorial Policy Checklist .

Statistics

For all statistical analyses, confirm that the following items are present in the figure legend, table legend, main text, or Methods section.

n/a	Confirmed	
☐	☒	The exact sample size (*n*) for each experimental group/condition, given as a discrete number and unit of measurement
☐	☒	A statement on whether measurements were taken from distinct samples or whether the same sample was measured repeatedly
☐	☒	The statistical test(s) used AND whether they are one- or two-sided<br>*Only common tests should be described solely by name; describe more complex techniques in the Methods section.*
☐	☒	A description of all covariates tested
☐	☒	A description of any assumptions or corrections, such as tests of normality and adjustment for multiple comparisons
☐	☒	A full description of the statistical parameters including central tendency (e.g. means) or other basic estimates (e.g. regression coefficient) AND variation (e.g. standard deviation) or associated estimates of uncertainty (e.g. confidence intervals)
☐	☒	For null hypothesis testing, the test statistic (e.g. *F*, *t*, *r*) with confidence intervals, effect sizes, degrees of freedom and *P* value noted<br>*Give P values as exact values whenever suitable.*
☒	☐	For Bayesian analysis, information on the choice of priors and Markov chain Monte Carlo settings
☒	☐	For hierarchical and complex designs, identification of the appropriate level for tests and full reporting of outcomes
☐	☒	Estimates of effect sizes (e.g. Cohen's *d*, Pearson's *r*), indicating how they were calculated

Our web collection on statistics for biologists contains articles on many of the points above.

Software and code

Policy information about availability of computer code

Data collection	For the data simulations we used an individual based model that we programmed in Python 2.7 using open source libraries such as Networkx and Numpy. The code for this simulation model will be made publicly available online, and further details are available on request. Simulations were run on the University of Bristol HPC cluster.
Data analysis	For the data analysis we used scripts written in R and Python with standard open source libraries. Specific libraries are detailed in the text where relevant. Details available on request.

For manuscripts utilizing custom algorithms or software that are central to the research but not yet described in published literature, software must be made available to editors/reviewers. We strongly encourage code deposition in a community repository (e.g. GitHub). See the Nature Research guidelines for submitting code & software for further information.

Data

Policy information about availability of data

All manuscripts must include a data availability statement. This statement should provide the following information, where applicable:

- Accession codes, unique identifiers, or web links for publicly available datasets
- A list of figures that have associated raw data
- A description of any restrictions on data availability

The scripts that support the findings of this study are available from the corresponding author on request. The simulation code will be made available online.

Field-specific reporting

Please select the one below that is the best fit for your research. If you are not sure, read the appropriate sections before making your selection.

Ecological, evolutionary & environmental sciences study design

All studies must disclose on these points even when the disclosure is negative.

Study description	A statistical ensemble of simulation runs of an individual based model of ecological community dynamics. We studied how community stability responded to habitat loss and the factors driving the changes in stability. 25 replicate simulations were run for each value of habitat loss, for 10 intensities of habitat loss between pristine landscape (0% loss) and 90% destruction. Simulations were repeated for 11 different fractions of mutualism to investigate the role of mutualistic interactions in mediating community responses.
Research sample	The data was derived from the ensemble of simulations described above. The model state was sampled at the end of each simulation run, and a number of descriptive variables used to calculate the relevant ecological metrics presented in the results.
Sampling strategy	We sampled the state of the simulation model directly. For example, species abundance was calculated exactly by summing the number of individuals of each species present in the landscape.
Data collection	Data simulated in silico.
Timing and spatial scale	The main ensemble of simulations took approximately three weeks to run on the HPC computing cluster at the University of Bristol. Subsequent simulations and sensitivity analysis were conducted between 2017 and 2019.
Data exclusions	No data were excluded.
Reproducibility	The simulation model is specified exactly in the publication such that it could be reproduced. Additionally the simulation code will be made available online for those wishing to reproduce our results.
Randomization	Not relevant as each parameterisation corresponds to a unique stochastic simulation run.
Blinding	Blinding not relevant to simulation study.

Did the study involve field work? ☐ Yes ☒ No

Reporting for specific materials, systems and methods

We require information from authors about some types of materials, experimental systems and methods used in many studies. Here, indicate whether each material, system or method listed is relevant to your study. If you are not sure if a list item applies to your research, read the appropriate section before selecting a response.

Materials & experimental systems

n/a	Involved in the study	
☒	☐	Antibodies
☒	☐	Eukaryotic cell lines
☒	☐	Palaeontology
☒	☐	Animals and other organisms
☒	☐	Human research participants
☒	☐	Clinical data

Methods

n/a	Involved in the study	
☒	☐	ChIP-seq
☒	☐	Flow cytometry
☒	☐	MRI-based neuroimaging
